# Echolocating Bats Cry Out Loud to Detect Their Prey

**DOI:** 10.1371/journal.pone.0002036

**Published:** 2008-04-30

**Authors:** Annemarie Surlykke, Elisabeth K. V. Kalko

**Affiliations:** 1 Institute of Biology, (SDU) University of Southern Denmark, Odense, Denmark; 2 Institute of Experimental Ecology, University of Ulm, Ulm, Germany; 3 Smithsonian Tropical Research Institute, Balboa, Panamá; Centre de Recherches su la Cognition Animale - Centre National de la Recherche Scientifique and Université Paul Sabatier, France

## Abstract

Echolocating bats have successfully exploited a broad range of habitats and prey. Much research has demonstrated how time-frequency structure of echolocation calls of different species is adapted to acoustic constraints of habitats and foraging behaviors. However, the intensity of bat calls has been largely neglected although intensity is a key factor determining echolocation range and interactions with other bats and prey. Differences in detection range, in turn, are thought to constitute a mechanism promoting resource partitioning among bats, which might be particularly important for the species-rich bat assemblages in the tropics. Here we present data on emitted intensities for 11 species from 5 families of insectivorous bats from Panamá hunting in open or background cluttered space or over water. We recorded all bats in their natural habitat in the field using a multi-microphone array coupled with photographic methods to assess the bats' position in space to estimate emitted call intensities. All species emitted intense search signals. Output intensity was reduced when closing in on background by 4–7 dB per halving of distance. Source levels of open space and edge space foragers (Emballonuridae, Mormoopidae, Molossidae, and Vespertilionidae) ranged between 122–134 dB SPL. The two Noctilionidae species hunting over water emitted the loudest signals recorded so far for any bat with average source levels of ca. 137 dB SPL and maximum levels above 140 dB SPL. In spite of this ten-fold variation in emitted intensity, estimates indicated, surprisingly, that detection distances for prey varied far less; bats emitting the highest intensities also emitted the highest frequencies, which are severely attenuated in air. Thus, our results suggest that bats within a local assemblage compensate for frequency dependent attenuation by adjusting the emitted intensity to achieve comparable detection distances for prey across species. We conclude that for bats with similar hunting habits, prey detection range represents a unifying constraint on the emitted intensity largely independent of call shape, body size, and close phylogenetic relationships.

## Introduction

Bats (Chiroptera) are the most ecologically diverse and, after the rodents, the second most speciose group of mammals (∼1100 spp.) [1]. Coupled with flight, the development of a complex echolocation system in all bats except flying foxes (Pteropodidae) is key to the successful radiation of this group permitting access to a broad range of resources at night [2]. Whereas new fossil findings suggest that flight evolved before echolocation [3], the subsequent evolution of a wide range of complex sonar systems across bats is unprecedented among mammals. Echolocation is used for orientation in space, and by many bats as the main sensory system for detection and localization of food [4]. Differences in call structure are linked to habitat type (cluttered versus uncluttered) and foraging mode (gleaning versus aerial hawking). They may also contribute to resource partitioning of sympatric species through fine-grained niche differentiation [5].

The range of echolocation depends on the intensity and frequency of the calls, the reflective properties of the surroundings and the sensitivity of the bats' hearing system [6]. Call intensity and frequency determine maximum detection distance for objects (obstacles, food) and range of acoustic interactions with other bats and hearing prey such as moths. Most bats are flexible in call design and adapt signal structure to environmental conditions. Handheld bats and individuals flying in confined spaces, for example a lab flight room, emit calls of shorter duration, larger bandwidth, and lower intensity than in open spaces [7]–[9]. Thus, biologically relevant data on echolocation calls must be measured from free-flying bats in the field. This is a challenge for sound intensity because of the high mobility of bats, their nocturnal lifestyle, the high directionality of their calls and the necessity to measure distance and direction between the microphone and individual bats.

Probably largely because of those difficulties, most field studies have concentrated only on frequency or temporal parameters of calls [10] with few including intensity [7], [11], [12]. *Eptesicus serotinus* [13] and *E. bottae* [14] flying in the wild emit signals with source levels (i.e. emitted intensity referenced to a standard distance of 10 cm from the bat's mouth) of 121–125 dB SPL. These data already suggest that call intensity for bats hunting insects in the air is far more intense than the standard source level of ca. 110 dB SPL for aerial hunting bats originally proposed by Griffin [15].

To measure source levels of bats in the field, and to test whether similar environmental conditions lead to similar sensorial adaptations, we studied a suite of sympatric aerial hawking and trawling bats on Barro Colorado Island in Panamá. The high diversity in call design across species and earlier studies on echolocation and foraging behavior of most species [16]–[20] provide crucial base-line data on variability in call structure, acoustic identification, and hunting habits. Accounting for ecological and phylogenetic variety, we studied 11 bat species from five families (Emballonuridae, Molossidae, Mormoopidae, Noctilionidae, and Vespertilionidae), representing all large families, except one, the Phyllostomidae, within the Yangochiroptera in the recent phylogeny given in [2] based on extensive molecular studies. The bats were hunting in open space, near vegetation or above water surfaces (i.e., background cluttered or edge space *sensu* [6]). Their main prey consisted of insects and was either caught in the air (aerial captures) or gaffed from the water surface (trawling).

We used a multi-microphone array and stereo-photography to determine the position of the bats for subsequent calculation of source levels of echolocation calls. We expected high source levels for search calls across taxa as all of the free-flying bats searched for prey in relatively similar acoustic environments. We also predicted variability in source level depending on distance to obstacles. Bats flying further away from obstacles may call louder than bats foraging closer to clutter-producing obstacles to enhance detection range. Trawling bats may benefit from the smooth water surface, which reflects most signal energy away from them. Furthermore, based on source level, we estimated maximum detection distances for prey across bat species within a local assemblage. We hypothesized that sympatric bat species should differ in maximum detection ranges based on different call intensity. Our results corroborated our expectations with respect to emitted intensity in that all bats emitted very intense signals. Contrary to our expectations, however, the maximum detection ranges for prey did not vary nearly as much as the emitted intensities. Finally, we discuss whether call intensity is likely to promote partitioning of acoustic space and hence co-existence of ecologically similar species and how it relates to their respective phylogenetic relationships.

## Results

### Echolocation calls and classification into functional groups

**Figure 1:**
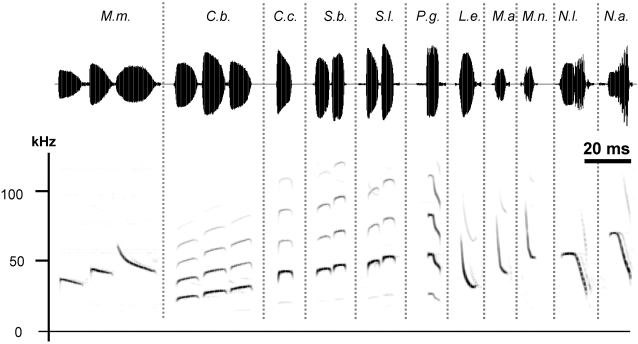
Echolocation calls. Time-signals and spectrograms of search phase echolocation calls of the 11 bat species studied. Dashed vertical lines separate species. The 20 ms time scale applies to both time signals and spectrograms, but pulse intervals between calls are collapsed. Calls from species emitting more than one call type were consecutive calls from one recording. *Molossus molossus (M. m.)* is an open air forager. *Cormura brevirostris (C. b.), Centronycteris centralis (C. c.), Saccopteryx bilineata (S. b.), S. leptura (S. l.), Pteronotus gymnonotus (P. g.), Lasiurus ega (L. e.), Myotis albescens (M. a.),* and *M. nigricans (M. n.)* are edge-space foragers. *Noctilio leporinus (N. l.)* and *N. albiventris (N. a.)* are trawling bats.

We estimated source levels for eleven species from five families, including 1) four species from the Emballonuridae (*Saccopteryx bilineata, S. leptura, Cormura brevirostris, Centronycteris centralis)*, 2) one Mormoopidae (*Pteronotus gymnonotus*), 3) one Molossidae (*Molossus molossus*), 4) two Noctilionidae (*Noctilio albiventris*, *N. leporinus*), and 5) three Vespertilionidae (*Lasiurus ega*, *Myotis albescens*, *M. nigricans*). Each bat emitted species-specific echolocation calls (Fig. 1) allowing unambiguous species identification [16], [18], [21]–[23]. Furthermore, calls revealed a strong phylogenetic component (i.e., similarity in general call shape within genera) including *(i)* the multi-harmonic call structure of the emballonurids, *(ii)* the pronounced second harmonic in the search calls of noctilionids and in the mormoopids, and *(iii)* the emphasis on the fundamental harmonic in the search calls of vespertilionids and molossids. Note the high frequency content and the number of higher harmonics of most calls exceeding previously reported values (Fig. 1) primarily because of the excellent signal-to-noise ratio of many recordings. We classified the bats into three functional groups according to their main foraging habitat and hunting behavior following [4]: 1) open space aerial forager (*M. molossus*); 2) edge space aerial foragers (all other species except *Noctilio*), and 3) edge space trawling foragers (both *Noctilio* species).

### Source levels across guilds

**Figure 2:**
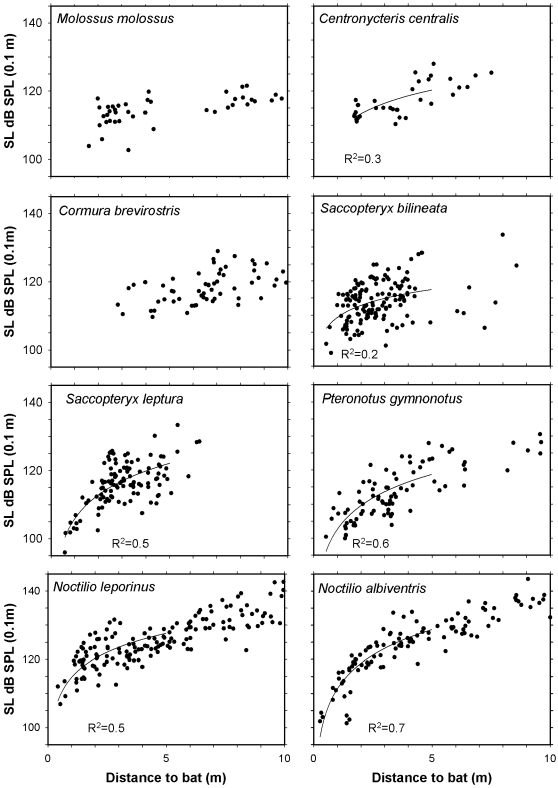
Echolocation call source levels. Scatter plots of estimated source levels (SL), i.e. emitted intensity in dB SPL 10 cm from the bat's mouth, as a function of distance between the bat and the array. Logarithmic trend lines (R^2^ values annotated) are shown for source level values as a function of distance at short distances, i.e. up to 5 m. Trend lines and R^2^ are only shown for those bat species, where correlations were statistically significant (P<0.001, t-test, [41]). The figure includes the eight species that were recorded over several nights.

**Figure 3:**
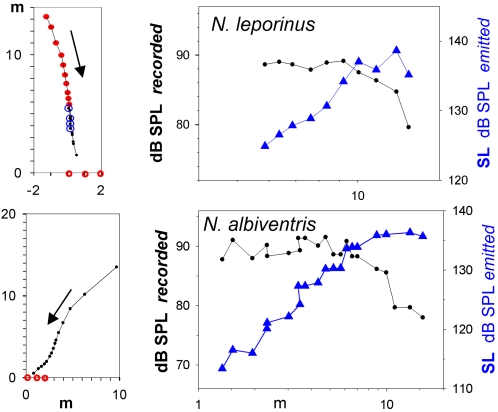
Measured call intensity and source level as bats approach the array. Two individual approach flights of *N. leporinus* (upper panel) and *N. albiventris* (lower panel). The flight paths (left panels) with arrows indicating the flight directions are shown as seen from above as the bats approached the three microphones (red circles on the x-axis). For *N. leporinus*, blue circles show positions based on photos. Source levels were estimated for the search calls marked by red in the flight path. The last calls in this recording were approach/terminal calls of a pursuit for which source level was not estimated. For *N. albiventris*, all calls were search calls and source levels were estimated for the whole sequence. The right panels show the recorded sound level and estimated source level (SL) as a function of distance between bat and microphone. At long distances the source level is constant, while at short distances the bat reduces the source level, such that the recorded level is constant as the bat gets closer.

Source level varied with species, but all bats emitted intense search calls (Fig. 2, Tab. 1). At close range there was a distinct correlation between distance and source level, such that the closer a bat was to the array and therefore also to the ground, vegetation or buildings behind the array, the less intense were its emitted calls (Figs. 2, 3). Source level increased by 4–7 dB/distance doubled (dd) (Tab. 1). At longer distances, the increase in source level leveled off and became independent of distance. Therefore we pooled the source level estimates in two distance groups, 0–5 m and 5–10 m depending on the distance from the array to the bat. Some bats may reduce output level even at distances above 5 m. Therefore even the 5–10 m group may include some distance-compensated calls, which may lead to an underestimate of the source level emitted in the search phase. Therefore table 1 also gives “Avg Max SL”, which is calculated as the mean of the maximum source level from each flight path. Since maximum source level is emitted at longer distances, we took “Avg Max SL” as the best representation of the emitted source level in search phase. The average maximum source level ranged from 121 to 137 dB SPL at long ranges (5–10 m) in the open well away from background clutter (Tab. 1). At longer distances positioning of the bat became less reliable. Thus, the data (Tab. 1, Fig. 2) do not include source level estimates from bats more than 10 m away from our array.

**Table 1:** Source levels

Bat	Frequency (maxE)	0–5 m		5–10 m		N
	kHz	Median SL (n)	dB/dd	Avg SL+SDev (n)	Avg MaxSL+SDev	
		*max			*max	
*M. m.*	36 43 46	113.7 (26) *120		118.0+2.2 (16)	120.6+1.7 *121.7	5
*C .b.*	25 28 31	114.1 (11) *119		120.5+3.2 (46)	120.5+3.2 *126.7	8
*C .c.*	43.5	115.0 (27) *125	4.3	123.7+2.7 (7)	123.3+3.0 *128.0	3
*S. b.*	45 48	114.2 (138) *126	4.0	121.8+7.3 (7)	127.3+5.8 *133.6	15
*S. l.*	52.5 55.5	116.4 (108) *130	6.8	126.8+4.6 (6)	126.8+4.6 *133.4	11
*P. g.*	55	110.3 (65) *128	6.2	123.9+3.7 (17)	126.7+3.4 *130.5	11
*L. e.*	32	106.9 (5) *117		117.5+3.3 (19)	120.7+2.7 *123.4	5
*M. a.*	43	105.7 (7) *111				2
*M. n.*	55	115.5 (7) *116		114.3+1.3 (6)	*116.4	1
*N. l.*	56	121.6 (79) *132	5.6	132.6+4.2 (26)	136.4+3.8 *142.7	9
*N. a.*	70	120.8 (67) *134	7.2	133.7+3.8 (39)	136.6+4.0 *143.5	8

The table shows source levels (SL) for search calls of 11 species of bats. Frequency(maxE) is the frequency with maximum energy in their calls. Source levels are pooled in two distance groups, 0–5 m and 5–10 m, according to the bat's distance from the microphone array. For 0–5 m, the table gives median values of all (n) source level estimates within this range. dB/dd is the increase in source level per distance doubled (dd) at short distances from trend lines in Fig. 2. For 5–10 m, the table gives two averages: Avg SL, the average of all (n) call estimates within this range, and Avg maxSL, average of the maximum source level from each approach flight (N). Overall maximum source levels at each distance range is shown with an asterisk: ^*^max. Averages and standard deviations were calculated from sound pressure levels in Pa, but given here as dB SPL. Thus, standard deviations are not symmetrical and only the positive SDev is given. For species abbreviations: see Fig. 1

### Source level within guilds

#### Open space aerial foragers

The only species in this group, *Molossus molossus*, started to forage at dusk, mostly hunting high above the ground in open space far (>7 m) from the vegetation, which limited the number of recordings within 10 m from the array. Structure of search calls was variable, including shallow-modulated signals interspersed with mixed signals composed of a steeper frequency-modulated (FM) element and a shallow component (Fig. 1). Interestingly, intensity seemed to be unaffected by the time-frequency structure as all call types had similar amplitudes across a recording (Fig. 1). Therefore, source level estimates for all call types were pooled. At short range median source level was 114 dB SPL. Due to the limited number of data the correlation between distance and source level at short distances was not obvious (Fig. 2). However, the increase in source level at long distance (121 dB SPL, Tab. 1), indicated that *M. molossus* reduced the output close to background.

#### Edge space aerial foragers

The eight species in this group revealed high variability in emitted intensity. At long range the average max. source level ranged from 121 to 127 dB SPL with absolute maximum values reaching up to 130–134 dB SPL. At short range, median estimates ranged from 106 to 116 dB SPL (Tab. 1).

Within this guild, *Cormura brevirostris* came closest to the open space aerial foragers as it foraged further away from vegetation than other species in this group. It was very active early in the evening and darted in fast flight, often at tree level or higher, across large tree-fall gaps, thereby keeping a distance of 5 m or more to the vegetation. As we did not obtain data from this species at close range (<2 m) we could not document a clear relation between distance and intensity. Overall, this species was among the least intense in this study with source level of 121 dB SPL at 5–10 m (Tab. 1).

The other edge space foragers hunted closer to vegetation and we got most recordings within 5 m distance. At 5–10 m there were few data and thus high variation in source level, in particular for *Saccopteryx bilineata*, with rather low average source level at 5–10 m (122 dB SPL), but with high maximum estimated source level (134 dB SPL). Most likely more recordings at longer distances would have yielded a higher average for this bat. Distance-source level relation was clear for short distances, but due to lack of long distance values it is unclear where distance compensation stops, possibly about 4 m.

The slightly smaller *S. leptura* resembled *S. bilineata* in flight and hunting behavior. Also for this species there was a lack of calls recorded at longer distances as they mainly flew within 5 m from obstacles. However, for short distances, there was a clear relationship between distance and source level (Fig. 2). Up to 5 m source level versus distance had a slope of 7 dB/dd (R^2^ = 0.5), but apart from a couple of intense source level estimates it seems that distance compensation levels off already at 2 m in this bat (Fig. 2). The average source level for search calls at distances above 5 m was 127 dB SPL (Tab. 1).

*Myotis nigricans* was only recorded once, but its flight path was nearly perfect, straight towards the array from 7 to 3.5 m. Over this range of distances, source level was quite constant around 116 dB SPL, indicating that *M. nigricans* starts reducing its output level closer than approximately 3.5 m from background. *Myotis albescens* was recorded in two good flight paths right after one another, thus probably representing the same individual. Seven calls were emitted while it was close (1.5–2 m) to the array and approaching it on a straight line. Due to the short distance the median source level of 106 dB SPL (max. 111 dB) is probably a very conservative estimate of emitted intensity. Based on distance compensation for the other bats (Tab. 1, Fig. 2). *M. albescens* may well emit source levels that are 10–15 dB more intense, when it is 5 m or more from background. *Lasiurus ega* was also only recorded on one night, but five flights were straight towards the array. Because these five recordings may be from the same individual, data analysis was kept to a minimum (Tab. 1). The maximum source level of 123 dB SPL was estimated from a call recorded from a bat 6 m from the array.

The maximum estimated source level for *C. centralis* was 128 dB SPL (Tab. 1). The data (Fig. 2) indicate that there was no distance compensation above 4 m. This species hunts almost exclusively in forest gaps.

*Pteronotus gymnonotus* was recorded every night. The extensive data base for this bat is based on search calls that are more evenly distributed over distances than the other bats in the guild. The slope of source level vs. distance was 6 dB/dd (R^2^ = 0.6) up to ca. 5 m, after which source level levelled out between 120 and 130 dB SPL (Fig. 2).

#### Edge space trawling foragers

The two trawling species, *Noctilio leporinus* and *N. albiventris*, were recorded in a small bay of Gatún lake. Both flew close and parallel to the water surface at low constant heights, mostly 0.2–0.4 m. The large number of high quality recordings permitted a robust data selection. The results confirmed our initial hypothesis about the specific (acoustic) conditions met when hunting over smooth water surfaces as both bats emitted very intense cries with average source levels of 136 and 137 dB SPL and maximum estimated levels above 140 dB SPL (Fig. 2, Tab. 2). For comparison, the level at a loud rock concert is 115–120 dB and for humans the threshold of pain is around 120 dB. Thus, the levels emitted by the *Noctilio sp.* are extremely loud; indeed the highest source levels that have been estimated to date for any free-flying bat in the field.

**Table 2:** Detection distances

Bat		*M.m.*	*C.b.*	*C.c.*	*S.b.*	*S.l.*	*P.g.*	*L.e.*	*N.l.*	*N.a.*
Bat	Mealworm	3	4	3	4	4	3	3	5	4
	small moth	6	10	7	8	7	6	7	8	6
	big moth	11	17	11	12	10	9	12	11	9
Prey	*moth threshold, dB SPL*	*50*	*45*	*54*	*54*	*57*	*60*	*58*	*60*	*66*
	detection distance	30	67	26	28	21	17	28	22	14

Estimated bat echolocation detection distances in m for a mealworm (target strength – 40 dB), a small moth (−20 dB), and a large moth (−5 dB) using the estimated maximum source levels for the nine bat species with sufficient data. Values for atmospheric attenuation [38] were taken at the most prominent frequency in the call spectrum for each bat (Tab. 1, Frequency(maxE)). All bats were assumed to have a detection threshold of 20 dB SPL. The eared prey's (moth) detection distances were estimated using average hearing threshold values for tropical nocturnal moths at the most prominent frequency emitted by each bat species (*Italics, moth thresholds* from [40]). For bat species abbreviations: see Fig. 1

Similar to most of the other species included in our study, *Noctilio sp.* showed a clear relation between distance and source level. The slopes at short distances were approximately the same in both species (6 dB/dd, R^2^ = 0.5) for *N. leporinus*, 7 dB/dd (R^2^ = 0.7) for *N. albiventris*) (Tab. 1, Fig. 2). To ensure that the slopes were not somehow a result of the data selection, we analysed our data for individual bats with regard to variations of estimated source level as a function of distance. Recordings with sequences of many cries emitted by bats heading towards the array typically showed that while the bat was still far away the recorded amplitude on the microphone increased as the bat approached. However, when the bat got closer than around 5–8 m, the recorded amplitude was constant, implying that the approaching bat reduced source level by ca. 6 dB per distance halved (Fig. 3), thus corroborating the slopes estimated from the pooled data.

**Figure 4:**
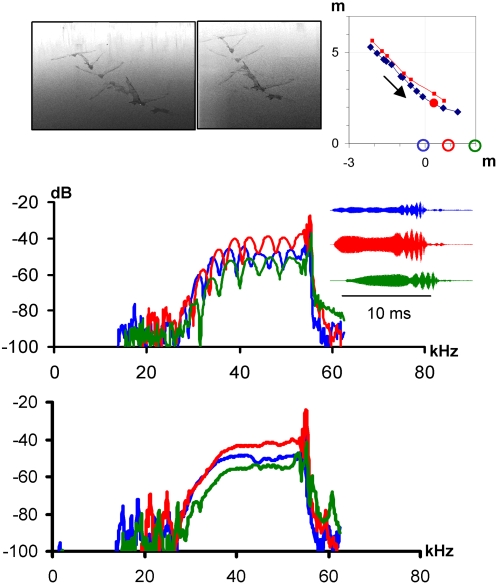
Removing interference from water reflections in echolocation calls. *N. leporinus* approached the array as shown by the multi-flash photos and reconstructed flight paths (seen from above) in the upper panel. The arrow indicates the flight direction towards the array with the three microphones, blue, red, and green circle, on the x-axis. The flight path based on photo-reconstructions (red) fits closely with the path based on sound (blue curve). The middle microphone was in the acoustic axis when the analyzed signal (indicated by a big red filled circle) was emitted. The color coded time signals illustrate how different simultaneous recordings of the same signal may be on the three microphones in the array. The color of time signals and spectra indicates the recording microphone shown by colored circles at 0, 1 and 2 m on the x-axis; blue: left microphone, red: middle microphone, green: right microphone. Spectra of recorded *N. leporinus* signals with notches from interference from water reflections are shown in the middle panel. The lower panel shows the effect of mathematically removing the reflections to leave only the smooth spectra of the emitted calls.

Recordings of bats flying over water revealed interference from the intense reflections of their sounds from the calm water [24] affecting source level estimates. We determined the reflection coefficient, α, and delay, ΔT, between direct and reflected signal. ΔT depends on distance and flight height. The measured ΔT-values revealed typical flight heights of 20–40 cm above water surface. In most cases α was between 0.9 and 0.95 (Fig. 4) indicating almost equal amplitudes of direct and reflected signals, which also means that the water itself reflects very little sound back to the bat, probably only creating minor background clutter problems in spite of the short distance. Thus, due to interference the peak amplitudes of the recorded time signals are up to double height (+6 dB) relative to the amplitudes of the direct signals.

### Detection distances

We estimated detection distances with a standardized set of three prey types: a small insect (mealworm head on), as well as a small and a large moth with target strengths of −40 dB, −20 dB, and −5 dB, respectively [25], [26] . Detection threshold was assumed to be 20 dB SPL for all bats and emitted search call intensities were assumed to be the maximum values estimated for long distances (Tab. 1). Surprisingly, our estimates showed that the range of detection distances is rather limited in spite of the large variations in source level within and across functional, phylogenetically distant groups of bats foraging in (acoustically) rather similar habitats. Max source level estimates varied more than 10-fold from 122 to 144 dB SPL, but estimated detection distances for a large moth varied only up to two-fold (Tab. 2). Remarkably, for *N. albiventris* emitting the most intense source level, estimates indicated that it had the shortest detection distances for both small and big moths (6 and 9 m) among all bats with sufficient data. Contrary to our expectations, *M. molossus* as a representative of open space aerial insectivores did not reveal the largest detection distances. Those were assigned to *C. brevirostris*. The source level for this bat was rather low, but its low frequency resulted in the longest detection distance estimates within the edge space aerial foragers, both for small and large moths.

Also, the range at which hearing prey can detect bats relies heavily on both intensity and frequency (Tab. 2). Even though prey insects usually have much higher hearing thresholds than bats, the prey's detection range is much longer than the bat's, because the prey detects the outgoing echolocation call, while the bat detects the weak reflected prey echo [25]. Again, it is not the most intense bats that may be detected furthest away, but *C. brevirostris*, where a combination of low atmospheric attenuation and lower hearing threshold for the prey resulted in estimated detection ranges of above 50 m.

## Discussion

Our results demonstrate that all of the eleven species of sympatric bats 1) emit very intense echolocation signals when hunting in their natural habitats, 2) adjust the emitted sound level of search calls to the distance to nearby obstacles and 3) converge on similar detection distances in spite of high variability in the intensity of their search calls, independent of their phylogenetic relationship.

### Evaluation of source level values

All bats in our study emitted average maximum source levels (121–137 dB SPL) clearly exceeding the level of around 110 dB SPL estimated by Griffin for high intensity aerial insectivores [15]. The source levels reported here even surpass those from the few newer field studies [13], [14]. Because of low sample size, source level estimates for the three vespertilionid species, *L. ega*, *M. albescens*, and *M. nigricans* should only be regarded as minimum values, but for the other eight bat species we conclude that our source level estimates provide realistic representations of the intensities emitted in search flight.

If anything, it is more likely that the estimates are too conservative (i.e. are underestimates), since bat echolocation signals are highly directional with maximum sound intensity only in the acoustic axis [27], [28]. This was the main reason for including only calls from bats flying towards the microphones, but even so bats may quickly turn their heads thus leading to off-axis recordings and hence under-estimation of source level. Data for *Eptesicus fuscus* [27], [28] indicate that at about 20 deg. off-axis the estimate would be 3–6 dB too low at 40 kHz.

In particular at long distances, source level estimate depends greatly on transmission loss, i.e. spherical spreading loss and atmospheric attenuation. Thus, any systematic error in estimating the distance to the bat would create errors in source level estimates. However, the close agreement between distances based on 3-D reconstructions from photographs and those estimated from the microphone array (Figs. 3,4) argues against this possibility, as does the fact that source level estimates levelled out at long distances corroborating results from free-flying vespertilionids [14]. There was a slight indication of continued increase of source level with distance for the two *Noctilio sp*. at long distances (Fig. 2). This is probably due to the sound quality criterion for selecting recordings for analysis. The further away the bat is the louder the calls had to be to reach criterion. Source level estimates for individual bats (Fig. 3) did not show this.

Source level was probably overestimated by about +6 dB for the two *Noctilio* due to positive interference from signal reflections on water surfaces [24]. However, we used these maximum source levels including positive interference for estimating detection distances, since interference will happen not only on the microphone, but also on the prey, and is indeed suggested as a reason why several bats from different families have evolved this hunting strategy [29].

### Bats adjust source level according to distance

Our data reveal distance compensation of source level at short distances for pooled data as well as for individual bats. Kick and Simmons [30] suggested, based on laboratory results, that *Eptesicus fuscus* keeps the output level constant, but the sensitivity in the ear improves with delay, such that better hearing threshold compensates precisely for the 12dB/dd that the echo level falls off with distance (automatic gain control, AGC). AGC was challenged by Hartley [31], [32], who found that bats reduce their output level by 6 dB per distance halved (dh) leaving 6 dB/dh for changes in auditory sensitivity. More naturalistic lab data with *Myotis daubentonii* suggested an intensity reduction of 3–4 dB/dh [8], corroborating our field data revealing slopes of −4 to −7 dB/dh. *Myotis daubentonii*'s upper limit to this behavior was not shown. *Eptesicus bottae* seems to stabilize the output level at distances exceeding 3 m [14], but our data indicate that many species compensate for distance up to at least 5 m. Distance compensation may be a general trend for echolocating animals as it has also been reported for toothed whales [33], [34]. It is likely that it is the array (and us) that the bats react to, but it may be buildings or other obstacles in the background. We did not pursue this further, since our focus was to get realistic source levels for search calls. Thus, details on distance compensation in the data were mainly established to avoid including estimates of source levels from short distances where output intensity is reduced.

### Detection ranges

Our results revealed several trends that deviate from our initial expectations. Firstly, it was surprising that the only bat flying in open space, *M. molossus*, did not produce the loudest calls nor did it reach the longest estimated detection distances. Its source level compared to the lower end of the edge space foragers. Although our results indicate that distance compensation ends at about 4 m, some loud calls may have been precluded from the data base, because we did not include calls from bats further away than 10 m. However, alternatively the relatively low source level is probably related to its small body size. Interestingly, source levels of the similar-sized *C. brevirostris*, an edge forager with a strong trend towards open space, were almost identical.

Secondly, the most intense source levels did not necessarily provide the longest prey detection ranges. For small targets, the maximum detection ranges of the two *Noctilio* species, which emitted the loudest calls exceeding all other intensity data known so far from free-flying bats, were still within the general range of all other study species. In fact, our estimates indicate that for large targets the two *Noctilio* had the shortest detection ranges, mainly due to the high frequencies of their search calls, where atmospheric attenuation is especially severe. Due to its lower call frequencies (25–31 kHz) *C. brevirostris* had the longest estimated maximum detection distance (10–17 m) among the bats studied.

This finding has important implications regarding the role of call structure for resource partitioning. If maximum prey detection ranges are rather similar among sympatric species, because bats compensate for increased atmospheric attenuation of high frequencies by increasing output intensity, then frequency may contribute less to fine-grained niche differentiation than previously assumed. Comparison of maximum detection ranges suggests that detection probabilities of small insects are indeed rather similar across bats. For larger prey, the pattern differs somewhat. Estimated detection ranges still vary much less than emitted intensities, but some bats achieve almost twice as long maximum detection distances as other, mainly due to lower emitted frequency. Thus, if call frequency was to play an important role in resource partitioning, we would expect dietary differences in the amount of large insects taken by the bats and less so in the amount of small insects. Those predictions need to be verified in behavioral experiments where factors such as food availability, food size as well as the bat's body size and condition can be controlled.

A similar pattern emerges for hearing prey, where our estimates indicate that they detect bats emitting lower frequencies further away than bats emitting higher intensities. Thus, a moderately sensitive insect will detect low frequency bats at distances up to 10 times the bat's detection distance for a medium sized moth, corroborating earlier results [25], while the same insect will only detect bats with high frequency calls at up to ca. 2 times the bat's detection distance (Tab. 2). This relation suggests another advantage of increasing emitted frequency in addition to the advantages usually mentioned, i.e. efficient reflection from small objects, and increased resolution.

Signal design in both *Noctilio* is probably mainly adapted for detection of minor disturbances on smooth water surfaces [18], [19]. Perhaps, the bats face a trade-off between improved target resolution requiring high frequencies and detection distance severely limited by atmospheric attenuation at high frequencies. As the smooth water surface acts as an acoustic mirror it reflects most signal energy away from the bat [29]. Thus, *Noctilio* can probably afford to call louder than in more cluttered environments and hence increase detection distance.

Thirdly, in spite of large variations in source level the prey detection distances did not vary nearly as much across species and families as one might expect given the high ecological diversity, call variability and size differences of the species sampled. Although maximum source levels varied by more than a factor of 10 the detection distances for all target strengths only varied by a factor 2–3 or less. Flight speed, background clutter, distance from ground and other species specific differences most likely also play a role in shaping echolocation call characteristics as does phylogenetic affiliation, but based on our results we conclude that sonar range is an important evolutionary constraint adapting call intensity to achieve approximately similar detection ranges for prey. This may be primarily linked to the fact that the study species all share similar habitats, where they either hunt flying insects in open or edge space or take them from the water surface. As acoustic properties of smooth water surfaces resemble edge and in part also open space, all eleven sympatric bat species are under comparable acoustic constraints while searching for their insect prey. The similarity in habitat may suggest that all bats share the same range of available insect prey, and thus it is likely that they have adapted their output intensity largely independently from phylogeny (i.e. particular features in call shape) to compensate for differences in atmospheric attenuation and to optimize prey detection.

Overall, our study underlines the importance of intensity measures in the field as source level plays a crucial and so far largely underestimated role in bat echolocation. If we want to further understand which ecological and evolutionary factors shape echolocation signal design, an even larger variety of call parameters need to be considered, including sound duration and pulse interval, which may create call-echo overlap or other masking effects. The basic assumption is that prey detection is only possible for prey at distances where its echo returns between the end of one pulse and the start of the next pulse. Comparing our data to detection distances for four emballonurid bats (*C. brevirostris, C. centralis* and both *Saccopteryx)* [16] revealed that they fall right within the call-to-call window for long and short range, supporting the importance of overlap-free detection. Finally, it is necessary to include other taxa, in particular members of the species-rich family of Neotropical leaf nosed bats (Phyllostomidae), which also form part of the Yangochiroptera, but forage mostly for stationary food including larger insects, small vertebrates, fruits, nectar, pollen and blood in different ecological settings within cluttered environments, i.e., forests, where food echoes often overlap with echolocation calls, to underline how our field based estimates of source level fit into this emerging larger picture of effect of all call parameters in the adaptation and evolution of echolocation.

## Materials and Methods

### Bats and study site

The study was conducted during wet season from September-October 1999, in November 2000, and from August-September 2003 on Barro Colorado Island (BCI), Panamá, a field station of the Smithsonian Tropical Research Institute (9 09′N, 79 51′W) near the Panamá Canal. Most of the recordings of free-flying bats were done at the forest edge close to the Old Dining Hall, a building on a slope about 100 m above Gatún lake. The two *Noctilio sp.* were recorded when they flew over the water. Recording sessions started when the bats began to forage, around half an hour after dusk (local sunset), and lasted until the activity became very low, usually around midnight.

### Sound recordings

**Figure 5:**
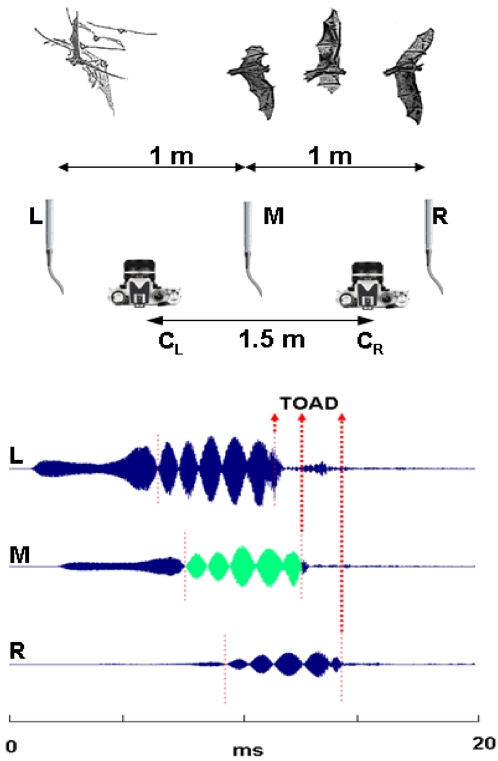
Methods for recording and photographing bats. Sounds were recorded with a linear microphone array with three microphones 1 m apart. The time-of-arrival-difference (TOAD) of sound on the microphones was determined by cross correlation between the three recordings using one (green part of the signal on M, middle microphone) as the model. TOADs were used to reconstruct flight paths. Some flight paths were also reconstructed from stereo photographs with a custom-made multiflash unit and two analogue 35-mm cameras (C_L_, C_R_). Flight paths from photographic reconstruction corroborated the positions based on array recordings. In the example shown here, the bat approached the left microphone before turning to the right (the bat's left) in front of the array. The echolocation call shown was emitted from a position 10 cm to the left of the left microphone, 1 m away from the array (x,y = −0.1, 1 m). Accordingly, the time-of-arrival differences show that the signal arrived first at the left microphone.

We used a combination of a linear microphone array with three microphones and stereo-photography composed of a multiflash unit with two cameras to estimate distance, direction and flight path of the echolocating bats (Fig. 5). The microphone array consisted of 3 G.R.A.S. ¼″ microphones (without grids) 1 m apart in a 2 m linear array. Sounds were amplified (G.R.A.S. 12AA, with custom built 13 kHz HP filter) and recorded digitally (sampling rate 250 kHz or 300 kHz per channel) using three channels on a Wavebook (IoTech) A/D with 128 MB circulating memory and stored on an IBM notebook computer. The frequency response of the whole recording chain was flat (within +/− 1 dB) from 13 kHz to 100 kHz. Most species were recorded with the array ca. 2 m above the ground with the microphones mounted on thin (5 mm) ca. 1 m long rods pointing slightly upwards. For recordings of the *Noctilio sp.*, the array was fastened with the microphones 0.6 m above the water.

In 1999, we recorded 400 sound files over 12 nights. In 2000, we recorded 118 files over 3 nights. In 2003, we focused on the two *Noctilio sp.* and recorded a total of 660 files over six nights. Only files with signals recorded at good signal-to-noise levels on all three microphones were included in the analysis. Flight paths were computed and source levels were estimated from 709 search calls (ranging from 24 to 145 calls for each species) emitted while the bats were flying towards the array. Some common bats, i.e. *S. leptura*, *S. bilineata, P. gymnonotus, M. molossus* and both *Noctilio* were recorded over many nights at several sites, so it is highly unlikely that all source level estimates were from the same individual.

### Stereo-photography

We photographed bats with a custom-made multiflash unit (Animal Physiology, University of Tübingen, Germany) with 12 flashes (Metz Megablitz CT45, guide number 50) using two analogue 35-mm cameras (Nikon 301, 35-mm lens). Release of cameras and flashes were triggered manually based on visual observations and bat detector output (for details see [19], [35]). Sounds of the discharge of the flashes as well as tape notes served to synchronize photos with the digital recordings on the IoTech Wavebook system.

### Data analysis

All recordings were sorted, first, on basis of sound quality, and secondly, after data processing, on basis of flight paths in order to find recordings of bats approaching the microphone on a direct course in order to select calls measured in the acoustic axis. The recordings were analyzed signal by signal in the frequency and time domain using a custom made signal analysis program, SigPro (Simon Boel Pedersen). The relative arrival times, and thus time-of-arrival differences of the sonar signals at the three microphones, were determined by cross-correlating the three recordings of each signal (Fig. 5). From the time-of-arrival differences for each pair of microphones we calculated a hyperbola defining the possible positions and the cross between the two hyperbolas was used as the estimate of the bat's position. The linear array technique yields only two of the three coordinates required for absolute positioning of a source. It places the source on a circle perpendicular to and with a centre on the line through the microphones. The location of the centre and the radius (distance to the bat) of the circle is given by the time-of-arrival differences [7], [36]. This is enough to estimate the source level, because that only requires knowledge of the distance to the bat (besides, of course, the recorded sound level), but in order only to analyze calls from bats approaching the array we determined the third dimension (e. g., above, behind, or in front of the array) from a combination of spoken comments and photos. All flight paths were determined from the acoustic measurements. Additionally, we made 3-D reconstructions of flight paths from multiflash photos from one night of recordings at the forest edge in 1999 and three nights of recordings over the water of *Noctilio sp.* in 2003. There was good agreement between the two methods both in terms of flight direction and absolute position of the bat. The comparisons between the two methods indicate that the accuracy of the linear array technique is usually better than 5% of the distance to the bat (i.e. 50 cm at 10 m), and never above 10% for recordings fulfilling the criteria of good signal-to-noise on all channels and flight paths approaching the array straight on. Due to the logarithmic nature of sound transmission loss with distance, lesser errors in distance would only have minor effect on the source level estimate. A worst case scenario would be a 1 m error at 10 m which would change the source level estimate for *N. leporinus* by at most 1.5 dB. Unless all distance errors were systematically biased to one side the effect on the average would be much less. Thus, we are confident that our results based on the array technique are reliable. Due to the post-triggering and long file duration, the acoustic method was much more efficient for data collection than the photographic method. Only recordings were the bats were in front of and approximately at the height of the array were included in the analyses. In some cases, the cross-correlation process gave delays that were obviously wrong. Signals with a very shallow sweep were quite sensitive to relative differences in Doppler shifts due to difference in the bat's relative velocity with respect to the three microphones. In particular, this was a problem for the *Noctilio* species where such shallow signals often alternated with signals with broader sweeps. In these cases, coarse time-of-arrival differences were estimated by comparing the start of the time signals on each channel to get a rough estimate of the bats' position at this particular signal and ensure it was the same bat.

Reflections created another problem for positioning and source level estimates, again in particular for the two trawling *Noctilio* species. The interference between direct and reflected signals created typical notches in the spectrum (Fig. 4, [24]). For numerical values the spectrum of (sound+reflection) on the microphone, *R_m_(f)*, is a function of *R(f)*, the spectrum of the direct echolocation sound, and α the reflection coefficient, and ΔT the delay between direct and reflected signal:
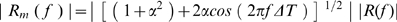



The reflections were removed mathematically (Program, “BatIron” developed by Simon Boel Pedersen, University of Southern Denmark) using the position and magnitude of the notches in the spectrum to determine the delay between direct and reflected signal and level of reflection from the water surface. Since the bat's distance and flight height determine ΔT, ΔT was used to assess flight heights. The compensation process was complicated by the fact that both α and ΔT varied throughout a signal. ΔT changed from beginning to end of a signal depending on the flight velocity of the bat and hence the relative position with respect to the microphone. α varied mainly because the direct and reflected signals were “seen” from different angles with respect to the bat. The reflected signal is recorded from an angle corresponding to a “virtual” microphone as far below the water surface as the real microphone is above the water surface. Due to directionality the intensity of the emitted signal is not the same on and off axis and this intensity difference is reflected in the deviation of α from 1, perfect reflection.

### Estimating source levels

Because bat emissions are highly directional, we had to select sonar calls emitted while the bats were flying directly towards one of the microphones in the array to assess the emitted intensity in the acoustic axis of the sound beam. The results (Table 1, Fig. 2) include only source level estimates based on search calls emitted at distances up to 10 m, because, as a rule-of-thumb, array based positioning is reliable at least up to 5 times the dimension of the array [36]. No late approach phase or terminal phase calls were included in these estimates. We used the recorded sound level, measured at the peak value of the signal and given as dB SPL re. 20 µPa rms, as well as the spherical spreading loss and the atmospheric attenuation to estimate the source level, i.e. the emitted sound pressure level at 10 cm's distance from the bat's mouth. We suspected that the spreading loss might be different from spherical −6 dB/dd, perhaps due to the water temperature and the time of night [37]. However, using an intense sound source (a “dog dazer” PetTrainer™ emitting a 26 kHz tone, 109 dB SPL at 1 m) we checked transmission loss up to 32 m and found no diversion from spherical spreading. We measured the frequency at the peak in each recorded call and used the atmospheric attenuation for that frequency to estimate transmission loss [38] for 28°C and 100% relative humidity, which corresponds to the average climatic conditions at the study site (for more details see [39]). The estimated source levels were used to calculate approximate detection distances for insect sized targets by using a simple form of the sonar equation:
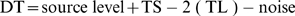
with SL: source level, TS: target strength, 2TL: two-way transmission loss from spherical spreading and atmospheric attenuation, and DT: detection threshold for the bat. The standard mammalian detection threshold is 0 dB under quiet conditions, and presumably higher for a flying bat. To take that into account and to include the noise term from the equation, for example background noise and wind noise, DT (including noise) was set to +20 dB. Detection distances for hearing insect prey were also estimated. Target strengths of moths, source levels and emitted frequencies of the bats in this study, as well as reported values for auditory thresholds for moths were used to estimate two-way and one-way transmission losses and thus detection ranges, see Table 1 and [25] for details.
